# Supplementary material for: Organizational models and patient-reported outcomes for palliative care across five tertiary hospitals in Nigeria: An environmental scan
Source: PLOS Glob Public Health. 2025 Jun 4;5(6):e0004638. doi: 10.1371/journal.pgph.0004638 (PMC12136337; doi:10.1371/journal.pgph.0004638)
Supplement: S3 Text — (DOCX) [file pgph.0004638.s003.docx]

Patient Responses

|  | Mean (SD) | | | | | |  |
| --- | --- | --- | --- | --- | --- | --- | --- |
| Item | Site 1  N = 13 | Site 2  N = 13 | Site 3  N = 13 | Site 4  N = 40 | Site 5  N = 53 | Overall^1^  N = 132 | p^2^ |
| POS 1 | 2.46 (1.51) | 2.15 (0.69) | 2.92 (1.61) | 2.30 (1.16) | 2.77 (1.22) | 2.52 (1.27) | .208 |
| POS 2 | 2.23 (1.36) | 1.15 (0.38) | 2.08 (1.55) | 1.73 (1.18) | 1.25 (0.48) | 1.69 (1.15) | **.001** |
| POS 3 | 2.77 (1.09) | 2.38 (1.04) | 2.38 (1.50) | 2.95 (1.41) | 2.98 (1.26) | 2.69 (1.27) | .394 |
| POS 4 | 4.46 (1.13) | 2.85 (1.41) | 4.08 (1.55) | 3.58 (1.13) | 4.19 (1.29) | 3.83 (1.39) | **.003** |
| POS 5 | 3.38 (1.19) | 2.54 (0.78) | 3.38 (1.80) | 3.25 (0.93) | 3.19 (1.14) | 3.15 (1.23) | .282 |
| POS 6 | 3.08 (1.19) | 3.38 (1.04) | 3.85 (1.46) | 3.20 (1.02) | 3.11 (1.14) | 3.32 (1.18) | .303 |
| POS 7 | 4.54 (1.20) | 3.77 (1.01) | 3.69 (1.49) | 3.80 (1.07) | 4.85 (0.36) | 4.13 (1.16) | **< .001** |
| ^1^ Equal weights across sites ^2^ ANOVA test | | | | | | | |

Items:

1. Please rate your pain during the last 3 days
   1. 1 = No pain, 5 = Worst/overwhelming pain
2. Have any other symptoms (e.g. nausea, coughing or constipation) been affecting how you feel in the last 3 days?
   1. 1 = Not at all, 5 = Overwhelmingly
3. Have you been feeling worried about your illness in the past 3 days?
   1. 1 = Not at all, 5 = Overwhelming worry
4. Over the past 3 days, have you been able to share how you are feeling with your family or friends?
   1. 1 = Not at all, 5 = Yes, I’ve talked freely
5. Over the past 3 days have you felt that life was worthwhile?
   1. 1 = Not at all, 5 = Yes, all the time
6. Over the past 3 days, have you felt at peace?
   1. 1 = Not at all, 5 = Yes, all the time
7. Have you had enough help and advice for your family to plan for the future?
   1. 1 = Not at all, 5 = As much as wanted

Caregiver Responses

|  | Mean (SD) | | | | | |  |
| --- | --- | --- | --- | --- | --- | --- | --- |
| Item | Site 1  N = 15 | Site 2  N = 13 | Site 3  N = 14 | Site 3  N = 14 | Site 4  N = 41 | Site 5  N = 53 | p^2^ |
| POS 8 | 4.33 (1.11) | 4.38 (1.04) | 3.93 (1.44) | 3.93 (1.44) | 4.00 (1.12) | 4.58 (0.84) | .064 |
| POS 9 | 4.47 (0.99) | 4.85 (0.38) | 4.21 (0.97) | 4.21 (0.97) | 3.56 (0.92) | 3.83 (0.89) | **< .001** |
| POS 10 | 2.47 (1.30) | 2.62 (1.26) | 3.21 (1.67) | 3.21 (1.67) | 3.41 (1.20) | 3.00 (1.27) | .098 |
| ^1^ Equal weights across sites  ^2^ ANOVA test | | | | | | | |

Items:

1. How much information have you and your family been given regarding the care you are receiving at this centre?
   1. 1 = None, 5 = As much as wanted
2. How confident does the family feel caring for the patient?
   1. 1 = Not at all, 5 = Very confident
3. Has the family been feeling worried about the patient over the last 3 days?
   1. 1 = Not at all, 5 = Severely worried
