# Supplementary material for: Organizational models and patient-reported outcomes for palliative care across five tertiary hospitals in Nigeria: An environmental scan
Source: PLOS Glob Public Health. 2025 Jun 4;5(6):e0004638. doi: 10.1371/journal.pgph.0004638 (PMC12136337; doi:10.1371/journal.pgph.0004638)
Supplement: S4A Text — (PDF) [file pgph.0004638.s004.pdf]

Admin/Lead Survey

AAA

Please complete the survey below.

Thank you!

Intervention Characteristics

Who is the head of your centre? Please provide name

Where is your site located compared to the main center?

- Onsite
- Offsite

Do you have a standalone centre, or are you affiliated with specific departments?

- Standalone
- Affiliated with departments

Where are PC services provided? Check all that apply.

- Outpatient clinic
- Inpatient - Consultative/primarily PC admissions
- Inpatient - Hospice Care
- Home visit

Outer Setting

Which of the following do you treat?

- Adults
- Children
- Both

Does the centre get referrals from other departments?

- Yes
- No

Inner Setting

When was the centre established?

M-D-Y

In total, how many staff are there at the centre?

How many nurses do you have at your centre?

How many physicians do you have at your centre?

How many social workers do you have at your centre?

How many pharmacists do you have at your centre?

What other professional cadre do you have in your PC centre?

Training

Does your centre or unit have Palliative Care seminars?

- ◯ Yes
- ◯ No

Does every staff member in the unit have PC training?

- ◯ Yes
- ◯ No

For those that are trained, was it formal or informal training?

We consider formal training to include certifications and fellowships.

We consider informal training to include workshops, seminars, shadowing, and other elective learning opportunities.

- ◯ Formal
- ◯ Informal
- ◯ Both

Do you have any certificate trainings/workshops planned for personnel in 2022-2023?

- ◯ Yes
- ◯ No

Do you have any plans for conference attendance by members or staff in 2022-2023?

- ◯ Yes
- ◯ No

Who do you go to if you have questions about Palliative Care?

Data Management and Research

Do you have a system in place to collecting data?

- ◯ Yes
- ◯ No

Has your centre began or published any research studies on PC?

- ◯ No, and we don't plan to
- ◯ No, but we would like to
- ◯ Yes

Centre Infrastructure

Do you have a room designated for pharmacy?

- ◯ Yes
- ◯ No

Do you have a room designated for medical records?

- ○ Yes
- ○ No

How many consulting rooms do you have in your clinic?

____

How many treatment rooms do you have in your clinic?

____

How many people can your waiting room seat?

____

Is your building space adequate?

- ○ Yes
- ○ No

Comment on your building space if you wish.

____

Do you have a housing system for patients' relatives?

- ○ Yes
- ○ No

Which of that following does your centre have? Select all that apply.

- ☐ Enrollment cards
- ☐ Tablets or computers for electronic record management
- ☐ Appointment system scheduler

Does your centre have case files with printed care questions for the following? Check all that apply.

- ☐ Social/Spiritual topics
- ☐ Plans of care
- ☐ Bereavement plans
- ☐ None of the above

Please rate how much you agree with the following statements.

		Disagree	Neutral	Agree	I don't know
1	I feel that our centre is well supported by the hospital.	○	○	○	○
2	I feel that we have adequate administrative support	○	○	○	○

Centre Funding

Does your centre make a profit from your services?

- ○ Yes
- ○ No
- ○ I don't know

Are any monetary allowances given to PC staff? If so, how much and how often?

Are staff paid by the institution or any other group?

Are you sponsored on this project?

- ◯ Yes
- ◯ No

Additional Comments

Is there anything else you would like to communicate about this subject of palliative care?

Submit

Powered by REDCap
