# Supplementary material for: Organizational models and patient-reported outcomes for palliative care across five tertiary hospitals in Nigeria: An environmental scan
Source: PLOS Glob Public Health. 2025 Jun 4;5(6):e0004638. doi: 10.1371/journal.pgph.0004638 (PMC12136337; doi:10.1371/journal.pgph.0004638)
Supplement: S4B Text — (PDF) [file pgph.0004638.s005.pdf]

Patient Questionnaire

A A A

Please complete the survey below.

Thank you!

Is anyone assisting you (the patient) with this survey?

○ Yes
○ No

Please rate your responses to the following prompts on a scale from 1 to 5.

	No pain<br>1	2	3	4	Overwhelming pain<br>5
Please rate your pain during the last 3 days	○	○	○	○	○

	Not at all<br>1	2	3	4	Overwhelmingly<br>5
Have any other symptoms (e.g. nausea, coughing or constipation) been affecting how you feel in the last 3 days?	○	○	○	○	○

	Not at all<br>1	2	3	4	Overwhelming worry<br>5
Have you been feeling worried about your illness in the past 3 days?	○	○	○	○	○

	Not at all<br>1	2	3	4	Yes, I've talked freely<br>5
Over the past 3 days, have you been able to share how you are feeling with your family or friends?	○	○	○	○	○

	Not at all<br>1	2	3	4	Yes, all the time<br>5
Over the past 3 days have you felt that life was worthwhile?	○	○	○	○	○

	Not at all<br>1	2	3	4	Yes, all the time<br>5
Over the past 3 days, have you felt at peace?	○	○	○	○	○

	Not at all<br>1	2	3	4	As much as wanted<br>5
Have you had enough help and advice for your family to plan for the future?	○	○	○	○	○

Submit

Powered by REDCap
