# Supplementary material for: Organizational models and patient-reported outcomes for palliative care across five tertiary hospitals in Nigeria: An environmental scan
Source: PLOS Glob Public Health. 2025 Jun 4;5(6):e0004638. doi: 10.1371/journal.pgph.0004638 (PMC12136337; doi:10.1371/journal.pgph.0004638)
Supplement: Attachment — Submitted filename: Response to Reviewers.docx [file pgph.0004638.s010.docx]

Reviewer One

| **Question** | **Comment** |
| --- | --- |
| My main overall piece of feedback is that the data analysis, particularly of the patient/caregiver surveys could be more robust. There is a mention early in the paper regarding a confidence interval used to select the sample size, but then only descriptive statistics are reported. Since the authors aim to compare models of care, it would be more interesting to compare outcomes across sites even if findings are not statistically significant. | Thank you for this comment. We agree that the sample size calculation description is irrelevant to the statistics we reported, so we have removed this at lines 170-172. See question 5 for our response about the sampling technique.  To address your point about the comparison between sites, we have included an additional table in the appendix (Appendix 3) comparing the mean (SD) ratings for each question by site, with ANOVA tests for equal means. We would like to point out that we did not aim to find associations between models of care and outcomes since the models are complex and it would require sampling many more sites. However, to your point, we did add some general observations we found when comparing the POS outcomes at the sites, including: 1) pain ratings at sites, (line 277-280) and 2) peace and life worthwhile ratings (lines 280-284). |
| Line 94: Typographical error: I think the authors meant "is expected to rise between 2020 and 2040" | This has been corrected. Line 94. |
| Line 96: What is the other side of this dual challenge? | 1)Rising NCDs and 2)limited health infrastructure. (Line 98). Punctation added to parse. |
| Line 135-136: The comment that all participated seems unnecessary unless other sites were invited who did not participate. | This statement has been deleted. (Lines 136-137) |
| 139 and 169: Please explain the sampling approach in more detail. How were participants selected? Was this a convenience sample or was random sampling used? Did any participants decline to participate? | A convenience sampling technique was used, and none of the participants declined to participate. This has been clarified at lines 175-176 |
| 162: This is a helpful explanation given the updates to CFIR. | Thank you |
| 167: Is there a reason no clinical or demographic information were collected? This may have helped to contextualize the findings. | This is a good question. After considering the complexity that a demographic and full clinical questionnaire would add to the data collection process, and the goals of the study, we elected not to collect demographic data and limit the clinical data to the African Palliative Outcome Scale (POS). (Appendix 3)We knew we would be sampling from small patient populations at each site, and we would not be powered to make appropriate statistical comparisons (such as through multiple regression) across patient and caregiver characteristics and the outcomes. We also believe not collecting this information made this study more feasible as we were working with limited resources and time. We do agree that this information would be meaningful and add context to the patient population. We have added this to the limitations and plan to collect this data in future, more robust studies. However, the sentence has been rephrased. (Lines 168-169) |
| 168: Since you didn’t include any statistical inferences and only descriptive statistics in the analysis, the mention of margin of error and confidence intervals does not seem relevant here. | Thank you, this has been deleted. (Lines 170-172) |
| 177: Could you say more about how the accuracy of the translations was ensured? Were the instruments translated ahead of time or translated in the moment by the research assistant? Was this person a trained translator? | Instruments were not pre-translated ahead of time; however, research assistants were selected from PC care staff who were accustomed to translating or discussing issues of PC with patients in their local languages. (Lines 183-186) |
| 183: Is there a reason you aggregated findings rather than comparing them between sites? Even if the sample size was small, this may have been useful information. | Per your suggestion, we have included an appendix table (Appendix 3) containing the patient and caregiver outcomes by site. See first comment response for details. |
| 184-185: Could you say more about the methodology of this qualitative rapid review? Were data coded? How were quotations selected? | We have updated the methods to explain this in more detail. This was added at lines 194-197  “Representative quotations were selected from frontline worker open-ended responses through a rapid qualitative review by two coders. Themes were identified for each response, and responses with unique themes were retained. If two responses contained identical themes, the response with the greatest clarity and detail was retained. Quotations were summarized in a table along with relevant CFIR 2.0 constructs.” |
| 196-197: Could you define the difference between PC admission and inpatient hospice care? | Inpatient PC services are consultative services, in which the patient is admitted by his or her primary clinician, who now refers to the PC unit for review or to co-manage, while PC admission is when the patient is admitted primarily by the PC physician for primarily PC needs. The definitions have been added as subscripts in Table 2 |
| Table 2: Could you clarify the difference between a standalone unit and a designated PC ward? | A stand-alone unit implies the PC service is provided as an independent functional unit within the hospital. Designated PC wards imply that the wards are designated only for patients with PC needs.  The definitions have been inserted as a subscript in Table 2. |
| 204-206: These ratios are somewhat hard to interpret. If I understood correctly, these same teams are involved in providing inpatient care? If so, the ratios are interesting but they don't take into account workload involved in inpatient care. | Thanks. This has been deleted. (line 225-228) |
| Table 3: Are all of these dedicated staff? Do they only work in the PC department/service or do they have other responsibilities? | This is a good point. No, they are not all dedicated staff, some do have other responsibilities in the hospital. We did not collect data on the exact hours spent by each staff providing palliative care services. We acknowledge this may skew staffing capacities and have added this to the limitations at line 371-373. |
| 219: I can see why including one leader per site was enough because you were focused on characteristics of the program and a single person could answer this. But a larger sample size for staff would have strengthened the findings. Perhaps you can offer an explanation for the small sample of staff (several sites only had 1 respondent). | This has been addressed in the limitations |
| 220-221: Interesting that this contradicts what leaders shared. Did they define training differently may  be? | Thank you for pointing this out. We recognized the lack of clarity and the statement has been rephrased appropriately to read as follows:  “One site (20%) reported that all staff members had some type of formal training in PC while the 4 other sites (80%) had some staff with formal training and others with informal training. (lines 237-241)” |
| Table 4: Did the participant mean empathic or empathetic? | Thank you. The response of the participant was quoted verbatim; hence it is difficult to say which the participant intended. Since both words have the same meaning we have retained the participant choice of word. |
| 237-244: It is difficult to interpret these proportions without a comparator. It may be more useful to compare these outcomes between sites for example. Did sites with different models of care have different outcomes? | This has been addressed in the first response above. We have included some comparisons as you describe, though we have done so with caution. |
| Additionally, when you say that 21% agreed they were in pain, was this a yes or no question? If not, it would be good to clarify if this includes patients who agreed as well as those who strongly agreed? And so forth for the other items. | We have clarified this in the text as follows:  “The following percentages reflect African Palliative Outcome Scale responses of 4 or 5 on the 5-point Likert scale with equal weighting by site (Figure 2). On average, sites found that 21% of their patients reported high levels of pain (POS 1)”  The updated language appears at lines 262-264(results) and 303-304 (discussion) |
| Does the question about pain assess if the participant is currently in pain or ever in pain? Does this indicate effective pain control or a population in which pain is not the primary problem? | Thank you for pointing out the lack of clarity. The description of the questions has been updated at line 165 to show that the questions ask for their experiences in the last 3 days:  “Patient and caregiver surveys aimed to reflect the quality of PC services through assessing patient-centered outcomes in the 3 days prior to survey collection”  And also in the Discussion at line 303-304:  “over 20% of patients on average reported high levels of pain (4 or 5 out of 5) in their last 3 days of care”  And is shown in the appendix questionnaires.  We believe this allows us to interpret in relation to pain control specifically. |
| Overall the analysis of the patient/caregivers surveys could be more robust. | See response to first comment regarding addition of appendix table with mean (SD) item ratings by site. |
| 251: It actually seems that pain control was quite good, although the finding is hard to interpret. | We have rephrased our discussion to include a paragraph entirely devoted to pain ratings and pharmacy staffing to clarify our interpretation of the results. Specifically, we mention that the ratings were better than expected, which we believe may be due 4/5 sites having a pharmacist on staff, but also highlight the fact that a considerable number of patients were reporting overwhelming pain, indicating room for improvement. (Lines 293-304) |
| 259: As I mentioned before, it’s hard to interpret this. If one nurse is seeing 10 outpatients in a week, that still seems like a good ratio, but it really depends on that nurse’s other duties. | This section has also been deleted since we did not capture the other responsibilities which the nurses and other staff have adequately. Lines 289-292 |
| 264: It would have been interesting to ask front-line staff and leaders specfiically about opioid access and use. | We agree this information is valuable. The next phase of our study included qualitative interviews that queried these issues in more detail. |
| 270: Could you address the discrepancy between how leaders and front-line staff described training coverage? | This has been addressed above. Lines 239-241 |
| 302-303: This is a helpful point about the need to incorporate spiritual support. | Thank you for this comment |
| 307-308: The phrasing here is a bit confusing. Positive in the sense that they agreed they had pain or positive in the sense that it was good that they had good pain control. Consider using "neutral or disagree" that they had been in pain. | Thank you for the comment. We have changed the wordings of positive to “favorable” and negative to “unfavorable”. Since agreeing is a good response for some items and a negative response for others, we believe this eliminates confusion.  Ex: line 295-297:  “most patients in our study reported neutral or favorable responses when asked if they had recently been in pain” |
| 312: This is a helpful notation of a limitation of the study. Consider mentioning that the question is ambiguous about whether this is assessing current pain, or "having been in pain" at some point. | See response to previous comment clarifying the question asks for pain in the last 3 days. |
| 335: Is this due to the number of sites or the number of participants per site? It seems as though you could compare for example, the patient centered outcomes between sites that had a standalone unit, different staffing ratios, or perhaps adequate space or not? | This limitation specifically relates to the number of features that comprise a care model and the number of sites included. Based on a previous comment, we did include some general observations (trend of pain outcomes and pharmacist on staff, peace outcomes and spiritual staff member). However, doing any quantitative analysis to test for associations with these features would require many sites or a more controlled setting, so we limited such conclusions to avoid overextending the scope of our data.  We acknowledge that it would very informative to be able to draw conclusions about how different aspects of the centres impact the patient-centered outcomes. However, this is a very broad question regarding a diverse set of PC environments, making it very difficult to assess quantitatively. We believe that the descriptive data in this paper provides important context for future research efforts that may uncover the ways we can improve PC in this environment, which would be largely qualitative. |
| 337: It seems as though your findings are more descriptive of the challenges different teams face more so than their model of care. I would have liked to hear more about how the programs were organized (standalone, outpatient, consulting) and how that relates to patient-centered outcomes. |  |
| 355: It would be helpful to mention ethics approval and consent procedures in more detail in the body of the manuscript | Thanks. This has been addressed. Line 200-209 |

Reviewer Two

| Question | My comment |
| --- | --- |
| The manuscript aims to answer 2 questions about palliative care delivery in Nigeria, namely 1. models of palliative care delivery and 2. patient and caregiver centred outcomes for those receiving palliative care. This manuscript adds to the literature and gives insight into palliative care development in Nigeria. | Thank you. |
| The authors main conclusions are justified by the data presented in the manuscript. | Thank you. |
| A weakness in this study is that only 5 out of 8 PC centres within tertiary health institutions were surveyed. The manuscript describes PC being provided in more than 20 sites in Nigeria, and it would have been interesting to learn more about organisational models also outside of tertiary centres. | This is a limitation, and we have thus mentioned our inability to generalize these results to other Nigerian centres in our limitations section. We agree that it will be both interesting and important to research a wider breadth of PC centres and providers in Nigeria. We hope to do so as we obtain additional resources and funding to continue this work. |
| Survey participants: The author describes the survey of frontline staff, yet the survey is not provided in the appendices.  The number of frontline staff surveyed was very low, and given one of the key reported findings was a lack of training in palliative care (only 56% of staff reported to have ever received palliative care training), the author should explain if efforts were made to survey more frontline staff and if not, why not. Also note discrepancy between leadership and frontline workers reporting of training. | Thanks for the comment. However, a summary was provided in appendix 2.  Frontline staff surveyed were senior PC staff who had a good grasp of the organizational models at their respective sites and information on staff training.  Thank you. We recognize this discrepancy in the levels of trainings received by staff. This has been rephrased for clarity as follows:  “One site (20%) reported that all staff members had some type of formal training in PC while the 4 other sites (80%) had some staff with formal training and others with informal training. (line 237-241)” |
| Data: The author provides data of organisational models in table 2, and the leadership survey (of organisational models) is provided in appendix 1. The data in table 2 misses some key information from the survey e.g. availability of home-based palliative care.  In addition, data is reported in table 2 that does not appear to be asked about in the survey included in the manuscript, e.g., housing system for patient’s relatives. | Thank you for your comment.  The missing data has been added to Table 2; all the centres offered Inpatient, outpatient and home-based care services.  The data was captured in the redcap survey but was omitted in the survey summary. This has been included (Appendix 1) |
| Staff ratios are reported 1:1 to 1:10 staff to patients per week, yet the location of care does not seem to be mentioned here (10 out-patients per staff member per week versus 10 in-patients?). | The result on staff ratios has been removed because it did not take into consideration whether the health workers were dedicated or worked only certain hours, which will hence provide biased data on staff strength and workload. |
| Interpretation of patient and caregiver POS: The author does not include in the manuscript the timing of patient and caregiver POS, e.g., on first assessment at time of referral, or 6 weeks after receiving palliative care etc. This is relevant to interpret the impact of palliative care on the POS. Yet the author appears to reach 2 contradictory interpretations from the POS in the discussion section. See Line 251/252 – highlight need to improve services for pain management and line 307/308 – suggests adequate pain control. | Timing:  Thank you for pointing out the lack of clarity. The description of the questions has been updated at line 163 to show that the questions ask for their experiences in the last 3 days: “Patient and caregiver surveys aimed to reflect the quality of PC services through assessing patient-centered outcomes in the 3 days prior to survey collection”  And also in the Discussion at line 303-304: “over 20% of patients on average reported high levels of pain (4 or 5 out of 5) in their last 3 days of care”  We believe this allows us to interpret in relation to pain control specifically.  Pain:  We have rephrased our discussion to include a paragraph entirely devoted to pain ratings and pharmacy staffing to clarify our interpretation of the results. Specifically, we mention that the ratings were better than expected, which we believe may be due 4/5 sites having a pharmacist on staff, but also highlight the fact that a considerable number of patients were reporting overwhelming pain, indicating room for improvement. Line 293-304 |
| Other comments: The author states in the introduction there are 3 million annual deaths in Nigeria, yet numbers of patients reviewed at each site are low (4 -20 patients per week). Does the author have any insights into why patient numbers are low despite the estimated need being so high? | PC care is in its nascent stage in Nigeria, and uptake is still poor and, hence, a low referral rate, as shown in Table 3. Only a smaller number than the actual population in need is attended to by the PC team. |
| Line 94 - PC estimated to rise to what? the figure is missing | This has been corrected. Line 94 |
